# High Monocyte-To-Lymphocyte Ratio Is Associated With Stroke-Associated Pneumonia

**DOI:** 10.3389/fneur.2020.575809

**Published:** 2020-10-06

**Authors:** Hao-Ran Cheng, Jia-Ying Song, Yi-Nuo Zhang, Yun-Bin Chen, Gang-Qiang Lin, Gui-Qian Huang, Jin-Cai He, Zhen Wang

**Affiliations:** ^1^Department of Neurology, The First Affiliated Hospital of Wenzhou Medical University, Wenzhou, China; ^2^School of Mental Health, Wenzhou Medical University, Wenzhou, China

**Keywords:** acute ischemic stroke, stroke-associated pneumonia, inflammation, monocyte, lymphocyte

## Abstract

**Purpose:** Stroke-associated pneumonia (SAP), a common complication in acute ischemic stroke (AIS) patients, is associated with poor prognosis after AIS. Inflammation plays an important role in the development of SAP. In this study, we aimed to explore the association between the monocyte-to-lymphocyte ratio (MLR) and SAP in AIS patients.

**Methods:** We continuously enrolled 972 AIS patients. SAP was diagnosed by two trained neurologists and confirmed by radiography, meeting the modified Centers for Disease Control and Prevention criteria. MLR values were measured for all participants, and all patients were evenly classified into three tertiles according to the MLR levels. We used the values that Youden's index max points corresponded to represent the optimal cutoffs, which represented the balance in sensitivity and specificity.

**Results:** 104 (10.7%) patients were diagnosed with SAP. SAP patients showed a significant increased (*P* < 0.001) MLR when compared with non-SAP. The optimal cutoff points of MLR were (T1) <0.2513, (T2) 0.2513–0.3843, and (T3) > 0.3843. The incidence of SAP was significantly higher in the third MLR tertile than the first and second MLR tertiles (21.7 vs. 4 vs. 6.5%, respectively, *P* < 0.001). After adjusting for confounding and risk factors, multivariate regression analysis showed that the third MLR tertile was an independent variable predicting the occurrence of SAP (odds ratio = 3.503, 95%CI = 1.066–11.515, *P* = 0.039).

**Conclusions:** Our study showed that higher MLR was significantly associated with SAP in AIS patients. MLR is beneficial for clinicians to recognize patients with a high risk of SAP at an early stage and is an effective way to improve clinical care of SAP patients. Higher MLR could be a helpful and valid biomarker for predicting SAP in clinical practice.

## Introduction

Stroke-associated pneumonia (SAP) is one of the most common complications after acute ischemic stroke (AIS), with an incidence of 6.7–36.98% (1–3), and may lead to lengthy hospitalization, poor functional outcome, and high morbidity and mortality (2, 4–7). It has been previously confirmed that the use of prophylactic antibiotics does not prevent SAP (8, 9). Due to the extra clinical and financial burden associated with SAP, it is necessary to explore underlying risk factors to aid with early recognition and prevention.

The SAP-related poor prognosis and several risk factors have been recognized in previous studies, creating several predictive models of SAP (5, 10–14). Hoffmann et al. developed and validated the A^2^DS^2^, a 10-point clinical model with high sensitivity and specificity for predicting SAP (3, 14, 15). This model assesses risk factors, including age, atrial fibrillation, dysphagia, sex, and previous stroke severity. In a total of 3,160 Chinese AIS patients, Li et al. (16) used machine learning methods to develop a model with high sensitivity and specificity to predict SAP. Interestingly, a study found that reduced vitamin D was a potential risk factor of SAP (17). In 2019, Zapata-Arriaza et al. (18) found that soluble urokinase plasminogen activator receptor and serum amyloid A, which were determined by immunoassays, were promising tools in early diagnosis of SAP. Advanced age, male, stroke severity, dysphagia, and low estimated glomerular filtration rate (eGFR) are predictive factors for SAP (5, 12, 13). In an animal experiment, C57BL/6 mice treated with an anti-CD147 antibody could decrease the lung damages, bacterial load, and pulmonary edema after receiving middle cerebral artery occlusion (19).

Evidence has found that inflammation is important in the development of SAP, and the associations between SAP and inflammatory biomarkers, such as interleukin 6, neutrophil-to-lymphocyte ratio, and C-reactive protein, have been explored (20, 21). Monocyte-to-lymphocyte ratio (MLR) is the absolute monocyte count divided by the absolute lymphocyte count and has been demonstrated to be a novel hematological and inflammatory parameter. MLR is associated with various diseases, such as community-acquired pneumonia, axial spondylarthritis, and coronary angiography, as well as the systemic inflammatory response, which reflects the abnormal immune status of diseases (22–24); however, the relationship between SAP and MLR remains unclear. In 2017, a study found that lymphocyte-to-monocyte ratios (LMRs) at admission were lower in AIS patients with pneumonia or urinary tract infection compared with patients without infections (25). Alternatively, the platelet-to-lymphocyte ratio (PLR) is the absolute platelet count divided by the absolute lymphocyte count, is a biomarker of systemic inflammation, and is related to the prognosis of hepatocellular carcinoma and the cognitive functions of breast cancer survivors (26, 27). Furthermore, leukocytes, monocytes, and lymphocytes have different roles in the inflammatory process, and their counts could directly reflect the inflammatory process. Meanwhile, the relationship between plasma biomarkers and AIS has been studied in many areas of stroke researches and found that biomarkers may help a lot in the early stage of AIS. Tu et al. (28) found that copeptin, as a plasma neuroendocrine biomarker, showed great ability in predicting a 3 month functional outcome and mortality after AIS.

To date, researches concerning the relationships between SAP and easy obtained blood biomarkers and comparing the predictive values of different biomarkers were insufficient. Our study could fill these gaps in previous studies and find the economical, objective, simple blood biomarkers. Thus, these biomarkers could help clinicians recognize AIS patients with a high risk of SAP at an early stage as well as reduce the financial and caring burden of patients. The purpose of the present study was to investigate the association between MLR and SAP in AIS patients, as well as find a helpful and valid biomarker for predicting and evaluating SAP in clinical practice.

## Materials and Methods

### Subjects

The present study was a retrospective study of patients with and without SAP. We obtained the approval of the Ethics Committee of the First Affiliated Hospital of Wenzhou Medical University (NO. 2019042) and conducted the study at the Neurology Department of the First Affiliated Hospital of Wenzhou Medical University under the Declaration of Helsinki.

**Figure 1:**
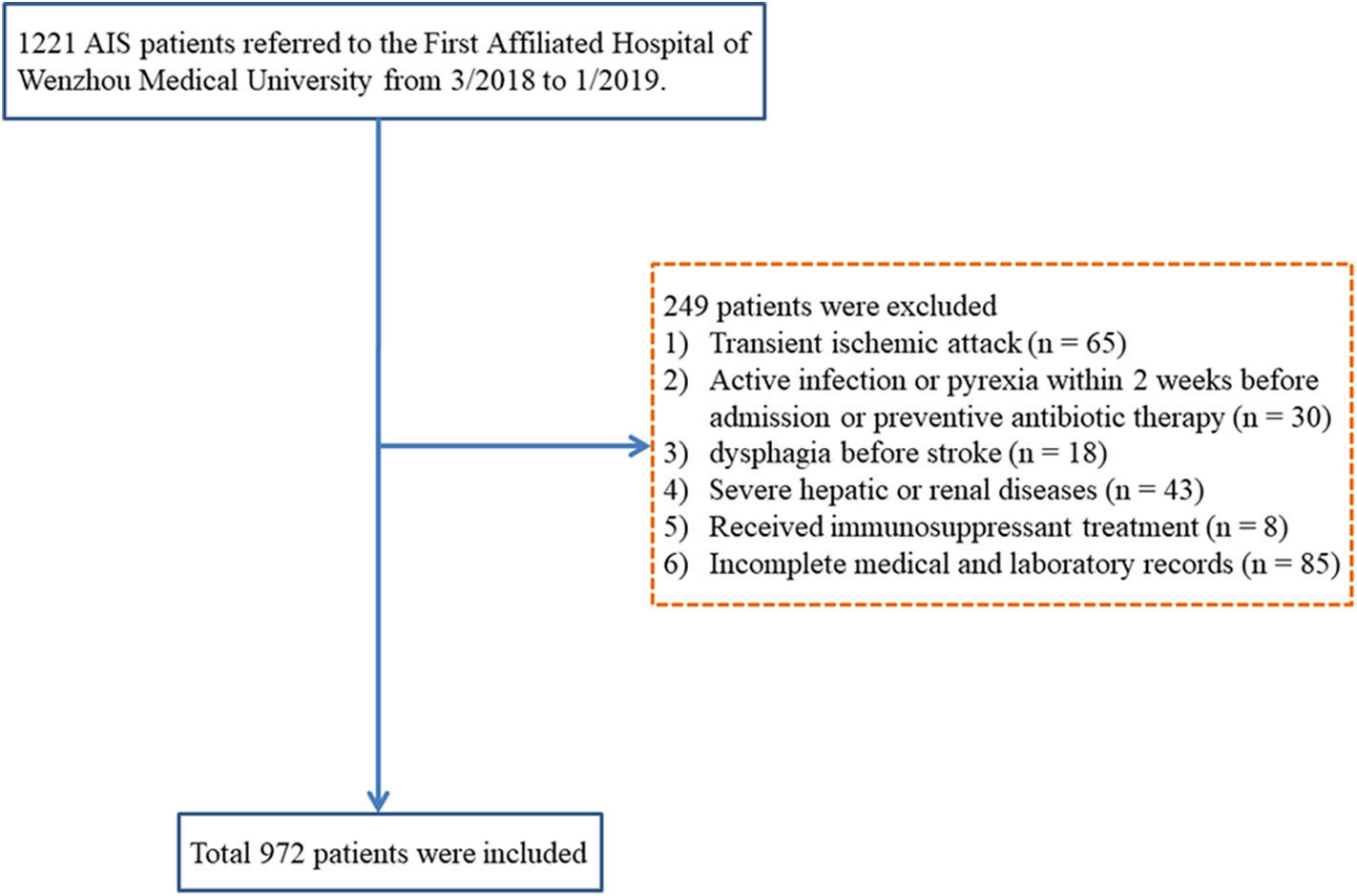
Research flowchart. AIS, acute ischemic stroke.

Patients, who were diagnosed with AIS and hospitalized in the Neurology Department of the First Affiliated Hospital of Wenzhou Medical University from March 2018 to January 2019, were continuously enrolled. The inclusion criteria were age ≥18 years and diagnosis of AIS confirmed by computerized tomography (CT) or magnetic resonance imaging at the time of admission. The exclusion criteria were transient ischemic attack; active infection or pyrexia within 2 weeks of admission or prophylactic antibacterial therapy; dysphagia before stroke; patients with any severe liver or kidney dysfunction; a history of the hematological disease, cancer, or received immunosuppressant treatment; pneumonia before stroke; or if patient's medical record was incomplete. A total of 972 AIS patients were enrolled in our present study (Figure 1).

### Diagnosis of Stroke-Associated Pneumonia

The diagnosis of SAP was based on the modified Center for Disease Control and Prevention criteria. SAP was defined as the infection of the lower respiratory tract during the first 7 days of hospitalization after stroke onset (20, 29), based on clinical symptoms, laboratory measurements, and confirmation via chest X-ray and CT (30). This study documented only hospital-acquired pneumonia and community-acquired pneumonia, and pneumonia before the stroke was not taken into consideration. SAP diagnosis was performed by two trained neurologists who were blinded to the patient's data.

### Data Collection

We collected demographic and clinical data retrospectively, including age, sex, history of previous disease, smoking, and drinking. Baseline blood pressure, thrombolytic therapy, stroke subtype (Trial of ORG 10172 in Acute Stroke Treatment criteria), and National Institutes of Health Stroke Scale (NIHSS) scores 24 h after admission were also recorded. Furthermore, all participants underwent the modified water swallowing test, a Japanese bedside dysphagia assessment, within 24 h after admission. In the modified water swallowing test, patients were asked to drink 30 mL water, and a trained neurologist would evaluate the swallowing reflex, wet hoarseness, and cough (31, 32). The existence of dysphagia, the use of mechanical ventilation, and nasogastric tubes were recorded to reflect swallowing functions. A^2^DS^2^ score was also rated for subsequent combined analysis. This score adds up scores of each item including age (≥75years, 1 point), atrial fibrillation (yes, 1 point), dysphagia (yes, 2 points), sex (male, 1 point), and stroke severity (0–4 NIHSS score, 0 points; 5–15 NIHSS score, 3 points; ≥16 NIHSS score, 5 points).

Within 24 h of hospital admission, blood from the antecubital vein was drawn and immediately sent for analysis. Laboratory parameters included red blood cells, leukocytes, neutrophils, monocytes, lymphocytes, platelets, fasting blood glucose, serum creatinine, and estimated glomerular filtration rate. All patients were evenly classified into three tertiles according to the MLR levels (tertile 1, <0.2513; tertile 2, 0.2513–0.3843; and tertile 3, >0.3843).

### Statistical Analysis

Continuous variables were analyzed using *t*-test or Mann–Whitney tests, presented as mean ± standard deviation or medians (quartiles). Categorical variables were compared using chi-square or Fisher's exact tests and denoted as relative frequencies and percentages. The Kruskal–Wallis test or one-way analysis of variance were used to compare the difference between three MLR tertiles in continuous variables, whereas Pearson's chi-square or Fisher's exact tests were used in categorical variables. Multiple logistic regression was used to adjust for potential confounding or risk factors. Receiver operating characteristics (ROC) curve analysis was used to determine the significant cutoff value, sensitivity, and specificity. The optimal cutoffs were values that Youden's index max points corresponded, which made the diagnostic effectiveness reach the best objectively and represent the balance in sensitivity and specificity (33). MLR and other biomarker values that were used to predict SAP were analyzed and compared using the area under the ROC curve (AUROC). *Z*-test was used for comparing ROC curves of MLR and other biomarker values (34). All statistical analyses used SPSS (version 19.0.0; IBM, Armonk, NY, USA) and MedCalc (version 13.0; MedCalc Software Ltd., Ostend, Belgium). Two-tailed *P*-values <0.05 were considered statistically significant.

## Results

### Baseline Characteristics of Acute Ischemic Stroke Patients Stratified by Stroke-Associated Pneumonia

During the research period, 972 AIS patients were included, and 104 (10.7%) patients were diagnosed with SAP (Figure 1). The baseline demographic, clinical, and laboratory variables are displayed in Table 1. A total of 619 (63.7%) patients were male, and the mean age of the enrolled patients was 67.0 years (59.0–74.0 years). Compared with non-SAP patients, SAP patients were older (*P* < 0.001), had higher NIHSS scores at admission (*P* < 0.001), were more likely to acquire dysphagia (*P* = 0.031), and were more likely to use nasogastric tubes (*P* = 0.017). For laboratory parameters, SAP patients had higher leukocyte counts, monocyte counts, MLR, and PLR, whereas non-SAP patients had lower lymphocyte counts. Furthermore, SAP patients demonstrated higher A^2^DS^2^ scores.

**Table 1:** Baseline characteristics of AIS patients stratified by SAP.

	**All patients**	**Non-SAP (*n* = 868)**	**SAP (*n* = 104)**	***P*-value**
Age (years), median (IQR)	67.0 (59.0–74.0)	66.0 (59.0–73.0)	72.0 (65.0–80.0)	<0.001
Male, *n* (%)	619 (63.7%)	553 (63.7%)	66 (63.5%)	0.960
Current smoking, *n* (%)	392 (40.3%)	349 (59.8%)	43 (41.3%)	0.823
Drinking, *n* (%)	361 (37.1%)	319 (63.2%)	42 (40.4%)	0.469
Baseline SBP (mmHg)	152.76 ± 24.34	152.50 ± 24.38	154.96 ± 23.94	0.330
Baseline DBP (mmHg), median (IQR)	82.00 (74.00–92.00)	82.00 (74.00–92.00)	83.00 (77.00–94.50)	0.194
NIHSS on admission, median (IQR)	3.0 (1.0–6.0)	2.5 (1.0–5.0)	9.5 (4.0–13.0)	<0.001
Previous stroke, *n* (%)	141 (14.5%)	121 (13.9%)	20 (19.2%)	0.148
Hypertension, *n* (%)	746 (76.7%)	665 (76.6%)	81 (77.9%)	0.095
Diabetes, *n* (%)	390 (40.1%)	356 (41.0%)	34 (32.7%)	0.855
CAD, *n* (%)	21 (2.2%)	21 (2.4%)	0 (0.0%)	0.155
AF, *n* (%)	118 (12.1%)	100 (11.5%)	18 (17.3%)	0.088
Thrombolysis, *n* (%)	38 (3.9%)	34 (3.9%)	4 (3.8%)	0.971
Dysphagia, n (%)	130 (13.4%)	109 (12.6%)	21 (20.2%)	0.031
Mechanical ventilation, *n* (%)	223 (22.9%)	198 (22.8%)	25 (24.0%)	0.783
Use of nasogastric tubes, *n* (%)	95 (9.8%)	78 (9.0%)	17 (16.3%)	0.017
A^2^DS^2^ score, median (IQR)	1.0 (1.0–3.0)	1.0 (1.0–3.0)	3.0 (1.0–5.0)	<0.001
RBC (×10^12^/L), median (IQR)	4.45 (4.14–4.77)	4.45 (4.16–4.77)	4.38 (3.98–4.68)	0.061
Leukocyte (×10^9^/L), median (IQR)	6.72 (5.57–8.26)	6.58 (5.50–7.92)	8.36 (6.32–11.05)	<0.001
Neutrophils (×10^9^/L), median (IQR)	4.24 (3.26–5.67)	4.14 (3.21–5.36)	6.15 (4.21–8.41)	<0.001
Monocyte (×10^9^/L), median (IQR)	0.51 (0.39–0.67)	0.50 (0.38–0.64)	0.68 (0.45–1.00)	<0.001
Lymphocyte (×10^9^/L), median (IQR)	1.64 (1.28–2.07)	1.67 (1.32–2.09)	1.30 (1.03–1.69)	<0.001
PLT (×109/L), median (IQR)	219.00 (182.00–257.50)	219.00 (184.00–257.50)	215.00 (171.25–258.75)	0.291
FBG (mmol/L), median (IQR)	5.60 (4.80–7.43)	5.60 (4.80–7.50)	5.80 (4.90–7.20)	0.788
Scr (μmol/L), median (IQR)	71.00 (59.00–84.00)	71.00 (59.00–84.00)	71.00 (58.00–85.00)	0.744
GFR, median (IQR)	90.90 (75.30–101.70)	91.15 (75.65–102.80)	86.60 (73.40–95.60)	0.015
MLR, median (IQR)	0.31 (0.23–0.43)	0.30 (0.22–0.41)	0.53 (0.33–0.71)	<0.001
PLR, median (IQR)	131.23 (102.58–172.27)	128.75 (100.95–168.66)	155.44 (127.03–208.18)	<0.001
Stroke etiology, *n* (%)				<0.001
Atherosclerosis	763 (78.5%)	693 (79.8%)	70 (67.3%)	
Cardioembolism	126 (13.0%)	96 (11.1%)	30 (28.8%)	
Small vessel occlusion	63 (6.5%)	60 (6.9%)	3 (2.9%)	
Other causes	20 (2.0%)	19 (2.2%)	1 (1.0%)	

*SBP, systolic blood pressure; DBP, diastolic blood pressure; NIHSS, National Institute of Health Stroke Scale; CAD, coronary artery disease; AF, atrial fibrillation; PLT, platelet; FBG, fasting blood glucose; GFR, glomerular filtration rate; MLR, monocyte-to-lymphocyte ratio; PLR, platelet-to-lymphocyte ratio*.

### Baseline Characteristics of Acute Ischemic Stroke Patients in Different Monocyte-to-Lymphocyte Ratio Tertiles

**Figure 2:**
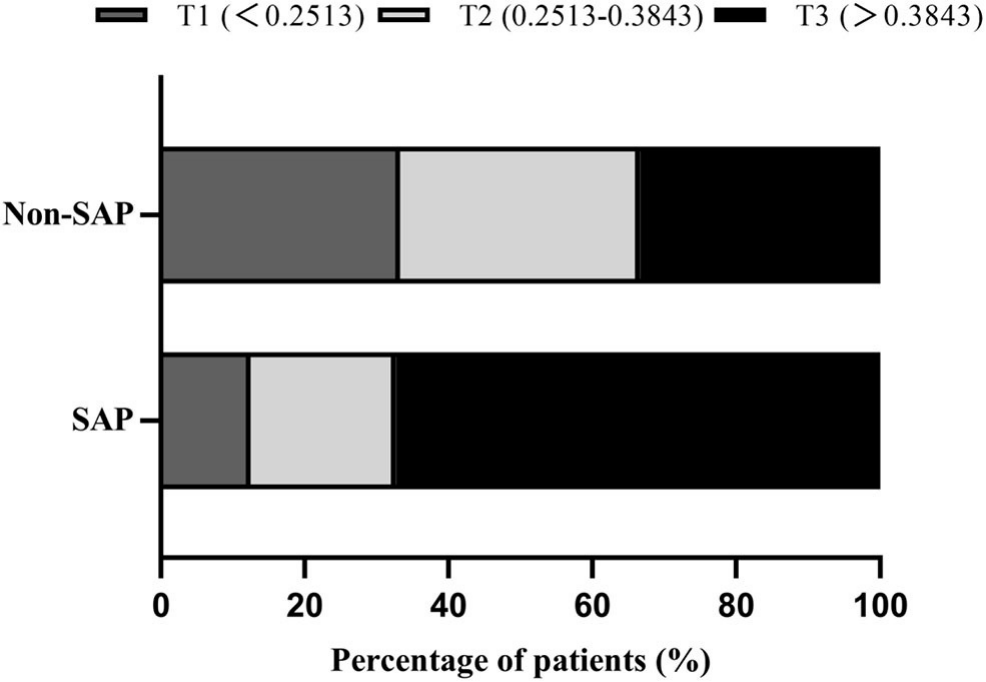
Percentage of SAP subcategorized groups by MLR tertiles in AIS patients. *F* = 32.941, *P* < 0.001.

Demographic, clinical, and laboratory variables, according to MLR tertiles, are depicted in Table 2. The incidence of SAP was significantly higher in the third MLR tertile than the first and second MLR tertiles (4 vs. 6.5 and 21.7%, respectively; *P* < 0.001; Figure 2). As shown in Table 2, patients with higher MLR levels were older; were more likely to be male, smoker, and diabetic; had higher NIHSS scores and A^2^DS^2^ scores at admission; and had higher leukocyte, monocyte, and PLR counts, as well as lower lymphocyte counts.

**Table 2:** Baseline characteristics of AIS patients in different MLR tertiles.

	**MLR tertiles**			
	**Tertile 1**	**Tertile 2**	**Tertile 3**	***P*-value**
	***n* = 323**	***n* = 323**	***n* = 322**	
	**(<0.2513)**	**(0.2513–0.3843)**	**(>0.3843)**	
SAP, *n* (%)	13 (4%)	21 (6.5%)	70 (21.7%)	<0.001
Age (years), median (IQR)	64.0 (58.0–70.0)	66.0 (58.0–73.0)	70.0 (62.0–79.0)	<0.001
Male, *n* (%)	165 (51.1%)	208 (64.4%)	243 (75.5%)	<0.001
Current smoking, *n* (%)	130 (40.2%)	128 (39.6%)	134 (41.6%)	0.871
Drinking, *n* (%)	121 (37.5%)	120 (37.2%)	120 (37.3%)	0.997
Baseline SBP (mmHg)	152.23 ± 22.89	154.55 ± 24.92	151.31 ± 25.08	0.218
Baseline DBP (mmHg), median (IQR)	82.00 (74.00–91.00)	82.00 (74.00–92.00)	83.00 (75.00–92.25)	0.699
NIHSS on admission, median (IQR)	2.0 (1.0–4.0)	3.0 (1.0–6.0)	4.0 (2.0-9.0)	<0.001
Previous Stroke, *n* (%)	47 (14.6%)	47 (14.6%)	47 (14.6%)	1.000
Hypertension, *n* (%)	258 (79.9%)	250 (77.4%)	234 (72.7%)	0.250
Diabetes, *n* (%)	159 (49.2%)	122 (37.8%)	107 (33.2%)	<0.001
CAD, *n* (%)	2 (0.6%)	10 (3.1%)	8 (2.5%)	0.070
AF, *n* (%)	32 (9.9%)	50 (15.5%)	36 (11.2%)	0.076
Thrombolysis, *n* (%)	16 (5.0%)	9 (2.8%)	13 (4.0%)	0.422
Dysphagia, *n* (%)	39 (12.1%)	42 (13.0%)	49 (15.2%)	0.485
Mechanical ventilation, *n* (%)	64 (19.8%)	84 (26.0%)	74 (23.0%)	0.166
Use of nasogastric tubes, *n* (%)	22 (6.8%)	34 (10.5%)	39 (12.1%)	0.067
A^2^DS^2^ score, median (IQR)	1.0 (0.0–2.0)	1.0 (1.0-3.0)	2.0 (1.0–4.0)	<0.001
RBC (×10^12^/L), median (IQR)	4.46 (4.16–4.77)	4.44 (4.17–4.74)	4.45 (4.06–4.80)	0.597
Leukocyte (×10^9^/L), median (IQR)	6.27 (5.29–7.43)	6.56 (5.47–7.91)	7.63 (5.95–0.90)	<0.001
Neutrophils (×10^9^/L), median (IQR)	3.64 (2.93–4.47)	4.28 (3.22–5.27)	5.41 (3.94–7.39)	<0.001
Monocyte (×10^9^/L), median (IQR)	0.38 (0.32–0.48)	0.51 (0.42–0.62)	0.70 (0.55–.086)	<0.001
Lymphocyte (×10^9^/L), median (IQR)	2.06 (1.68–2.46)	1.66 (1.39–2.01)	1.27 (1.00–1.59)	<0.001
PLT (×109/L), median (IQR)	228.00 (187.00–263.00)	219.00 (186.00–254.00)	211.50 (171.00–252.75)	0.134
FBG (mmol/L), median (IQR)	5.50 (4.70–7.40)	5.60 (4.80–7.75)	5.80 (4.90–7.50)	0.304
Scr (μmol/L), median (IQR)	66.00 (57.00–78.00)	72.00 (58.00–85.00)	73.00 (61.50–90.00)	0.113
GFR, median (IQR)	92.70 (80.20–103.20)	89.70 (75.80–102.00)	88.00 (70.95–100.40)	0.001
MLR, median (IQR)	0.20 (0.17–0.23)	0.31 (0.28–0.35)	0.51 (0.43–0.65)	<0.001
PLR, median (IQR)	108.16 (88.24–133.16)	130.40 (105.65–165.63)	163.09 (129.84–214.51)	<0.001
Stroke etiology, *n* (%)				<0.001
Atherosclerosis	270 (83.6%)	264 (81.7%)	225 (69.9%)	
Cardioembolism	17 (5.3%)	28 (8.7%)	81 (25.2%)	
Small vessel occlusion	26 (8.0%)	25 (7.7%)	12 (3.7%)	
Other causes	10 (3.1%)	6 (1.8%)	4 (1.2%)	

*SBP, systolic blood pressure; DBP, diastolic blood pressure; NIHSS, National Institute of Health Stroke Scale; CAD, coronary artery disease; AF, atrial fibrillation; PLT, platelet; FBG, fasting blood glucose; GFR, glomerular filtration rate; MLR, monocyte-to-lymphocyte ratio; PLR, platelet-to-lymphocyte ratio*.

### Association Between Monocyte-to-Lymphocyte Ratio Levels and Stroke-Associated Pneumonia

In the univariate logistic regression, continuous MLR values was associated with SAP (odds ratio [OR] = 1.487, 95% confidence interval [CI] = 1.359–1.627, *P* < 0.001). In the multivariate logistic regression, the occurrence of SAP was used as a dependent variable, and the first MLR tertile was used as a reference for all AIS patients. Multivariate regression analysis showed that the third MLR tertile was an independent variable for predicting the occurrence of SAP (OR = 6.624, 95%CI = 3.582–12.250, *P* < 0.001; Table 3). These differences remained significant after adjusting for confounding and risk factors (Model 1: OR = 5.156, 95%CI = 2.207–12.042, *P* < 0.001; Model 2: OR = 3.503, 95%CI = 1.066–11.515, *P* = 0.039; Table 3).

**Table 3:** Multivariate logistic regression analysis of clinical determinants of SAP in AIS patients.

	**Unadjusted**		**Model 1**		**Model 2**	
	**OR (95%CI)**	***P*-value**	**OR (95%CI)**	***P*-value**	**OR (95%CI)**	***P*-value**
MLR tertiles						
Tertile 1	Reference		Reference		Reference	
Tertile 2	1.658 (0.816–3.371)	0.162	1.438 (0.564–3.668)	0.447	1.643 (0.463–5.836)	0.443
Tertile 3	6.624 (3.582–12.250)	<0.001	5.156 (2.207–12.042)	<0.001	3.503 (1.066–11.515)	0.039

*MLR, monocyte-to-lymphocyte ratio; AF, atrial fibrillation; NIHSS, National Institute of Health Stroke Scale; GFR, glomerular filtration rate. Model 1: adjusted for age, sex, current smoking, hypertension, diabetes, and AF. Model 2: adjusted for covariates from Model 1 and further adjusted for NIHSS on admission, dysphagia, use of nasogastric tubes, and GFR*.

**Figure 3:**
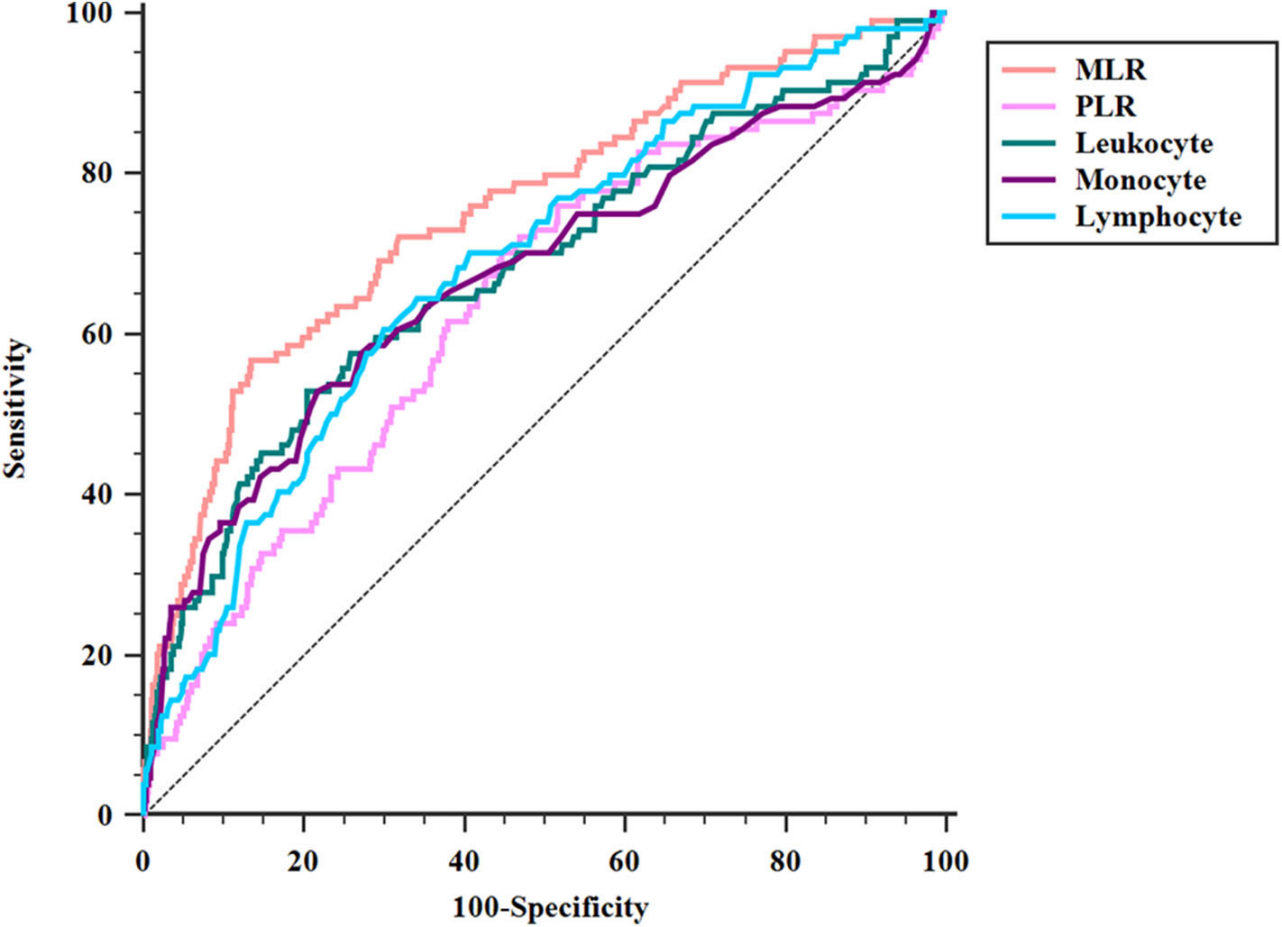
Comparison of area under the receiver operating characteristic curve (AUROC) values among MLR and biomarkers of SAP. MLR vs. PLR, *P* < 0.001; MLR vs. leukocyte, *P* = 0.017; MLR vs. monocyte, *P* < 0.001; MLR vs. lymphocyte, *P* = 0.012.

**Figure 4:**
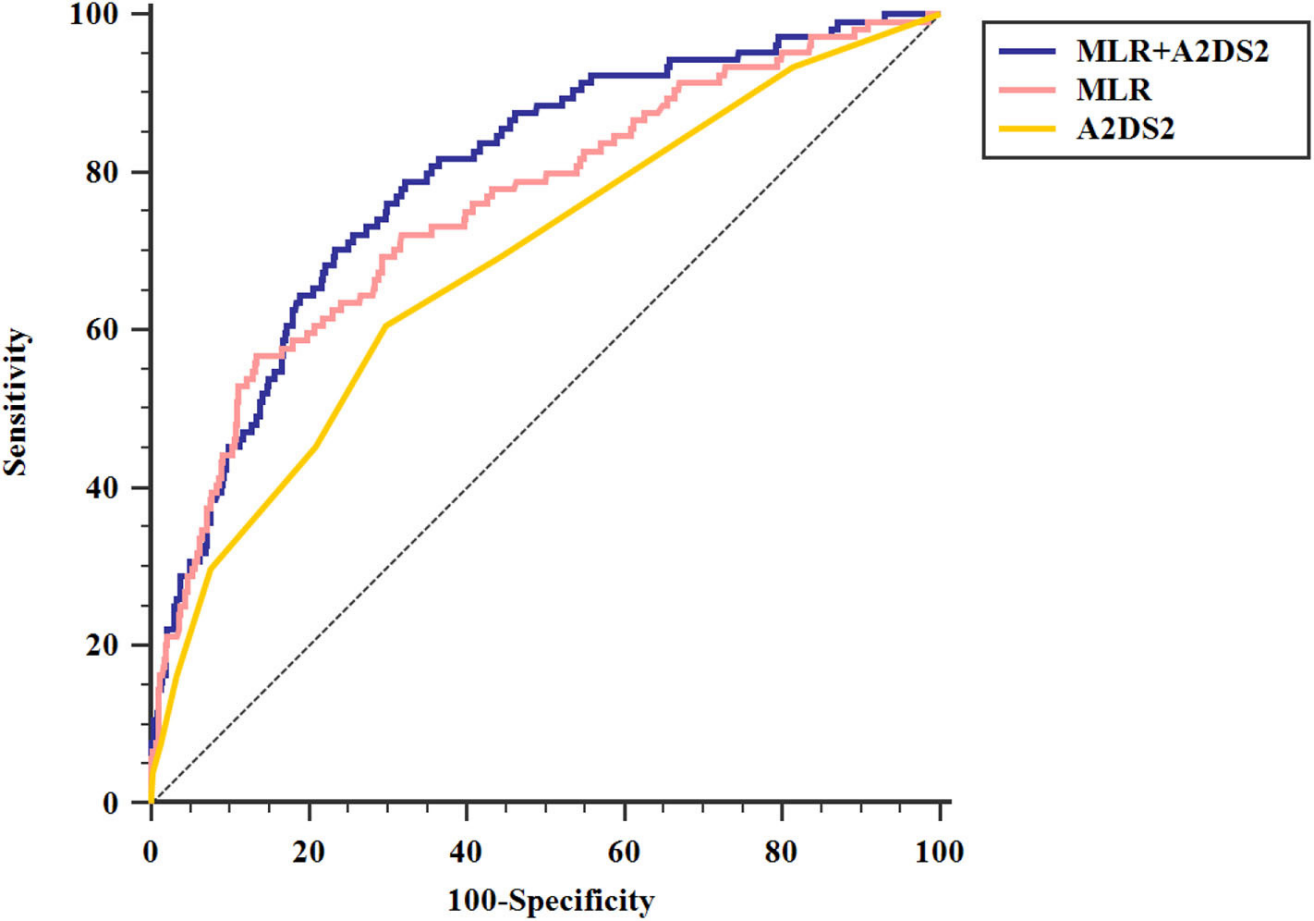
Comparison of area under the receiver operating characteristic curve (AUROC) values among A2DS2 score and MLR combined with A2DS2. MLR+ A2DS2 vs. MLR, *P* = 0.032; MLR+ A2DS2 vs. A2DS2, *P* < 0.001.

ROC analysis showed that the optimal MLR cutoff score for SAP was 0.49 with 0.756 (95%CI = 0.727–0.782) AUROC, 56.73% sensitivity, 86.57% specificity, 33.7% positive predicted value, and 94.2% negative predicted value (Table 4, Figure 3). Compared with other biomarkers, the predictive value of MLR was higher than PLR (AUROC = 0.637, 95%CI = 0.606–0.667; Table 4), leukocyte counts (AUROC = 0.681, 95%CI = 0.651–0.711; Table 4), monocyte counts (AUROC = 0.673, 95%CI = 0.643–0.703; Table 4), and lymphocyte counts (AUROC = 0.686, 95%CI = 0.656–0.715; Table 4). All differences remained significant when MLR was compared with other biomarkers (Figure 3). The AUROC of A^2^DS^2^ score was 0.685 (95%CI = 0.656–0.715; Table 4, Figure 4), whereas the AUROC of the modified A^2^DS^2^ score, which combined A^2^DS^2^ score with MLR, was 0.794 (95%CI = 0.767–0.819; Table 4, Figure 4). The difference between the modified A^2^DS^2^ scores and MLR (*P* = 0.032, Figure 4) was statistically significant, whereas the difference between the modified A^2^DS^2^ score and A^2^DS^2^ score (*P* < 0.001, Figure 4) was also statistically significant.

**Table 4:** Results of ROC analysis about biomarkers with SAP.

	**AUC**	**Cutoff**	**Youden index**	**Sensitivity (%)**	**Specificity (%)**	**PPV (%)**	**NPV (%)**	***P*-value**
MLR	0.756	0.49	0.433	56.73	86.57	33.7	94.2	<0.0001
PLR	0.637	134.47	0.255	70.19	55.32	15.9	93.9	<0.0001
Leukocyte	0.681	8.31	0.323	52.88	79.42	23.6	93.3	<0.0001
Monocyte	0.673	0.67	0.311	52.88	78.24	22.6	93.2	<0.0001
Lymphocyte	0.686	1.41	0.306	60.58	70.02	19.6	93.7	<0.0001
A^2^DS^2^	0.685	2.50	0.309	60.58	70.28	19.6	93.7	<0.0001
MLR+ A^2^DS^2^	0.794	0.12	0.468	70.19	76.62	26.5	95.5	<0.0001

*MLR, monocyte-to-lymphocyte ratio; PLR, platelet-to-lymphocyte ratio*.

## Discussion

To our knowledge, ours was the first study to explore the relationship between MLR and SAP. We found that higher MLR was significantly associated with the prevalence of SAP in AIS patients. Additionally, we demonstrated that MLR combined with the A^2^DS^2^ model could effectively predict the incidence of SAP. MLR would be an economical, objective, simple blood biomarker to help clinicians recognize AIS patients with a high risk of SAP at an early stage as well as reduce the financial and caring burden of patients. Therefore, higher MLR could be a helpful and valid biomarker for predicting and evaluating SAP in clinical practice.

Compared with the current literature, our study firstly described the relationship between MLR and SAP and compared the discriminating power of SAP in different biomarkers and the modified A^2^DS^2^ score. In our study, 104 (10.7%) patients were diagnosed with SAP, which was consistent with the previous studies (1–3). We found that AIS patients with older age, higher stroke severity, and dysphagia were more likely to acquire SAP, which was also consistent with the previous studies (5). Furthermore, our study added the treatments, including mechanical ventilation and nasogastric tubes, into the analysis and found that AIS patients who used nasogastric tubes were more likely to acquire SAP. Although nasogastric tubes were used to prevent aspiration in AIS patients, several studies described that prolonged use of nasogastric tube was related to an increased incidence of SAP (35, 36). Researchers explained that nasogastric tubes might lead to the dysfunction of the esophageal sphincters and desensitization of the pharyngoglottal adduction reflex (36). Also, we found that SAP patients had lower levels of GFR compared with non-SAP patients, which had not been studied yet.

MLR has been demonstrated as a novel hematological parameter in several medical fields. The elevated MLR level may be a result of the increased monocyte counts or/and decreased lymphocyte counts. In the present study, the MLR levels of SAP patients were higher than non-SAP patients (0.53 vs. 0.30, *P* < 0.001; Table 1), and the MLR level of all AIS patients was higher than the non-stroke control patients in a previous study of axial spondyloarthritis (0.31 vs. 0.20, respectively; Table 1) (24). This could be attributed to the inflammatory response led by the occurrence of stroke. Immune cells would infiltrate into the infarction area and then trigger the accumulation and production of chemokines and inflammatory cytokines (37, 38); however, the association between MLR and AIS requires further study.

The relationship between MLR and SAP has not been fully studied; however, there are potential mechanisms, such as inflammatory recruitment and immunological suppression after AIS. Monocyte is important in the initiation of inflammatory processes and is a sentinel and effector of infection (39). During inflammation, monocytes increase and recruit to the site of inflammation, change into terminally differentiated cells, and promote the renewal of dendritic cells and tissue macrophages (39, 40). In a previous study, MLR was confirmed to be a predictive factor in the diagnosis of *Klebsiella pneumoniae* infection (41). Another study performed by Huang et al. (23) showed that higher MLR was significantly associated with community-acquired pneumonia. Thus, increased MLR levels may suggest the initiation of inflammatory processes, which is congruent with previous reports.

Stroke-induced immunodepression syndrome (SIDS) is an immunological change in patients after stroke, and growing evidence has suggested that SIDS could protect our body from secondary inflammatory injury after stroke but would increase the susceptibility to SAP in AIS patients (42, 43). When a stroke occurs, brain vessels would be blocked, which subsequently results in hypoxia, nutrient deficiency, and metabolic product accumulation in the infarction area. Thus, a stroke could lead to local inflammatory response and result in SIDS (44). Xabier et al. (45) found that patients with stroke-associated infection had higher monocyte counts. Additionally, Hoffmann et al. (44) found that reduced monocytic human leukocyte antigen-DR (HLA-DR), led by SIDS, could predict SAP independently. During the novel coronavirus 2019 pandemic, a new study found that decrease depression of monocytic HLA-DR in critically ill patients may lead to immunosuppression (46). Thus, we hypothesized that SIDS would decrease the expression of monocytic HLA-DR and compensatively increase monocyte counts. Meanwhile, SIDS could result in a rapid decrease of peripheral blood lymphocyte counts and functional inactivation of T cells (47). Konstantin et al. (48) found that lymphocytes suffered a comprehensive apoptotic loss after stroke, and a catecholamine-mediated lymphocytic defect was essential for stroke-associated infection. Meanwhile, Park et al. (25) found that lower LMR on day 7 is associated with worse outcomes at 3 months in AIS patients and suggested that LMR may be a helpful biomarker for indicating SIDS for the features of SIDS are decreased of lymphocyte counts and deactivation of monocytes. Our study found that high MLR at admission was associated with the occurrence of SAP, which added and supported their hypothesis of SIDS marker in the acute phase of AIS. Therefore, SIDS could influence the monocyte and lymphocyte counts, which could explain the association between MLR and SAP.

In our study, we also made comparisons between MLR and several cell counts, PLR and A^2^DS^2^ model. MLR and PLR were reported to be independent risk factors in many infectious diseases (49). Also, the counts of leukocytes, monocytes, and lymphocytes could directly reflect the inflammatory and immunity process. Among these biomarkers, MLR was the best biomarker for predicting SAP in our study. Stroke, as major stress for the human body, would trigger reactions to this stressful event such as inflammatory and immune responses after onset. However, there were a small number of researches that studied the associations between SAP and easily obtained biomarkers of inflammatory and immunity and compared their predictive values. Our study filled these gaps in previous studies and found that MLR was an economical, objective, and simple parameter to predict SAP in an early stage. Hoffmann et al. (14) developed the A^2^DS^2^ scoring system to predict the risk of SAP, which has validated in different cohorts (3, 14, 15). In a valid external comparison, Zapata-Arriaza et al. (15) found that A^2^DS^2^ had the highest sensitivity (87%) and specificity (92.8%), compared with the AIS-associated pneumonia score and the pre-stroke Independence, Sex, Age, National Institutes of Health Stroke Scale. Our current study described that the SAP patients had a higher A^2^DS^2^ score than non-SAP patients, which was consistent with the prediction model. Also, we found that the modified A^2^DS^2^ score, which combined the A^2^DS^2^ score with MLR, had the highest discriminating power of SAP, compared with the A^2^DS^2^ score (*P* < 0.001, Figure 4) or MLR (*P* = 0.032, Figure 4). The AUROC of the modified A^2^DS^2^ score (0.794, Table 4) was also higher than the A^2^DS^2^ score (0.685, Table 4) and MLR (0.756, Table 4). As the indexes of the A^2^DS^2^ score only included demographic and clinical parameters at admission, it was developed for use on the first day after stroke onset. Thus, it cannot reflect the inflammatory process in time. Although MLR could reflect the inflammatory response in AIS patients, it could also modify the A^2^DS^2^ score.

There are some limitations to our current study. First, this was a single-center and retrospective study; therefore, we cannot establish causality between MLR and SAP, necessitating the use of future multicenter, prospective studies. Secondly, MLR was only measured at admission and unable to be measured at other time points (beyond 24 h), so we cannot evaluate the association between MLR and SAP dynamically. In further studies, we would measure MLR at multiple times during the hospitalization to investigate whether a high MLR is developing only after stroke in certain patients or is this an interindividual difference that patients already present with independent of the stroke. Thirdly, the newest inflammatory factors, such as interleukin-6, were not included in this study. Further studies could record more inflammatory factors to clearly explain the relationship between inflammation and pneumonia.

In conclusion, the present study showed that higher MLR was significantly associated with SAP in AIS patients. When combined with A^2^DS^2^ scores, the newly developed model had a higher discriminating power of SAP. MLR is beneficial for clinicians to recognize patients with a high risk of SAP at an early stage and is an effective way to improve clinical care of SAP patients. Therefore, higher MLR could be a helpful and valid biomarker for predicting SAP in clinical practice.

## Data Availability Statement

The data are not publicly available due to privacy or ethical restrictions. Requests to access these datasets should be directed to Zhen Wang, wangzhen@wzhospital.cn.

## Ethics Statement

The studies involving human participants were reviewed and approved by the Ethics Committee of First Affiliated Hospital of Wenzhou Medical University. Written informed consent for participation was not required for this study in accordance with the national legislation and the institutional requirements.

## Author Contributions

H-RC: conceptualization, data curation, formal analysis, and writing the original draft. J-YS, Y-NZ, and Y-BC: data curation and formal analysis. G-QL: data curation. G-QH: conceptualization and project administration. J-CH: conceptualization, resources, and writing-review and editing. ZW: funding acquisition, resources, and writing-review, and editing. All authors contributed to the article and approved the submitted version.

## Conflict of Interest

The authors declare that the research was conducted in the absence of any commercial or financial relationships that could be construed as a potential conflict of interest.
